# In vivo oscillations of the soleus muscle can be quantified using b-mode ultrasound imaging during walking and running in humans

**DOI:** 10.1038/s41598-020-77266-w

**Published:** 2020-11-19

**Authors:** A. K. M. Lai, E. F. Hodson-Tole

**Affiliations:** 1 Department of Biomedical Physiology and Kinesiology, Simon Fraser University, Burnaby, Canada; 2 Department of Life Science, Musculoskeletal Science and Sports Medicine Research Centre, Manchester Metropolitan University, Manchester, UK

**Keywords:** Musculoskeletal system, Image processing, Physiology

## Abstract

Impact forces, due to the foot contacting the ground during locomotion, can be considered input signals to the body that must be dissipated to prevent impact-related injuries. One proposed mechanism employed by the body to damp the impact is through vibrations of the skeletal muscles. However, there is yet to be direct in vivo measures of muscle oscillations during locomotion. This study investigated the use of 2D ultrasound imaging to quantify transverse muscle oscillations (deep-superficial displacement of the muscle boundary relative to the skin) in response to impact forces elicited by walking and running at a range of speeds. Increases in vertical impact forces with faster walking and running was consistent with changes in both magnitude and frequency in the measured oscillations of the soleus muscle; one of the main human ankle plantar flexors. Muscle oscillations contained more higher frequency components at fast running (50% signal power in frequencies below ~ 14 Hz) compared with slow walking (50% signal power contained in frequencies below ~ 5 Hz). This study provides a platform for ultrasound imaging to examine muscle oscillation responses to impact forces induced by changes in external interfaces such as shoe material, locomotion type and ground surface properties.

## Introduction

Impact force, caused by interactions with the external environment such as the foot colliding with the ground during running, can be considered input signals that travel through the body and must be properly damped or muscle fatigue and incidences of impact-related injuries could occur^1^. Although adjusting joint stiffness is commonly linked with dissipating the input signal^2,3^, vibration or oscillation of soft-tissues in the body, such as skeletal muscle, have also been suggested to significantly contribute to damping them and the corresponding energy^1,4,5^. For example, previous studies have used computer models of wobbling masses, skin-mounted accelerometers and grids painted onto the skin and found that the wobble of soft-tissues can assist in dissipating input signals and energy during locomotion^6–8^. Furthermore, studies that have combined skin-mounted accelerometers and measured muscle activity have shown that muscles have the capacity to modulate their activity, and hence oscillation characteristics, in response to impact force^4,9,10^. However, while we know that skeletal muscles are important for attenuating these ground impact shock waves, and that soft-tissue oscillations (or wobble) play a role in this attenuation, we know relatively little about the features of these oscillations.

Current measurements of soft-tissue responses to an external stimulus have been limited to measuring oscillations at the skin interface. Skin motion artifacts can decouple motions observed at the skin from the underlying soft tissue^11,12^ and can also contain multiple components of accelerations (rotational, translational, gravitational) that may be influenced by impact forces making it difficult to interpret^13^. Thus, there is currently no direct method of quantifying how skeletal muscles oscillate in response to foot–ground impacts during locomotion, meaning we are poorly equipped to understand how they contribute to dissipating the input signal. This may be particularly important for understanding behaviours of deeper muscles, whose movements are less likely to be related to skin movement given their greater distance from the skin surface.

Ultrasound imaging has been used extensively to examine in vivo muscle behaviour during dynamic movements^14–16^. Typical quantities that are extracted from ultrasound images include muscle fascicle length and velocity, muscle thickness and fascicle pennation angle. A large proportion of ultrasound studies of muscle behaviour in humans have focused on the main ankle plantar flexors, soleus (SO), medial gastrocnemius (MG) and lateral gastrocnemius (LG)^17^. This is largely due to their importance for locomotion, location on the leg (which means the transducer can be attached with limited interference to gait) and pennate fascicle architecture (which means fascicle orientation, strain and strain rate can be robustly quantified)^17^. Additionally, due to their location in the distal leg, these muscles can be considered good candidates to illustrate the influence of ground impacts on in vivo muscle oscillations^7^. Dynamic ultrasound imaging can also be used to quantify the behaviour of the aponeurosis that typically spans the surface of the muscle belly^18,19^. The behaviour of the aponeuroses can be used to represent the boundaries of the muscle shape. Quantifying displacement of the aponeurosis could be used to provide direct measurements of transverse (deep-superficial) and/or longitudinal (distal–proximal) oscillations that may occur in the imaged muscle without being observed at the skin surface and thus, if measured, could help provide new insights into muscle behaviours associated with impact force dissipation.

In this study, we examined the feasibility of using dynamic 2D ultrasound imaging of the deep soleus (SO) muscle, to measure how transverse oscillations, induced by ground impact forces, vary with increased walking and running speeds. Specifically, we hypothesised that (1) the higher ground impact force with faster steady-state walking and running speeds will induce greater input signal to the body, and (2) as locomotion speed increases there will be an increase in the power and frequency content of the in vivo oscillations within the superficial and deep aponeuroses of the SO during stance phase.

## Methods

### Experimental protocol

Experimental data of walking and running were taken from ten participants (9M/1F; age, 27 ± 6 years; height 1.81 ± 0.08 m; mass 80.2 ± 11.7 kg) as part of a larger study investigating in vivo muscle function during locomotion^14^. Participants gave their informed consent and none suffered from any pre-existing neuromuscular injuries at the time of testing. Ethics approval was granted by the local committee at University of Queensland, where data were collected (Ref. No.: 2012001215), and all experiments were performed in accordance with relevant guidelines and regulations.

The participants were asked to walk and run on a treadmill at three steady-state walking speeds (0.7, 1.4 and 2 m s^−1^) and four running speeds (2, 3, 4, and 5 m s^−1^). All participants were allowed to self-select their foot strike pattern, stride frequency and stride length. Foot strike patterns were determined using two criteria: initial ankle plantar-flexion angle and the presence of a ground impact peak^20,21^, both of which were manually determined. Three participants chose a rear-foot strike pattern for all running conditions, three participants chose a mid-foot strike pattern for all running conditions and four participants shifted to a mid-foot strike pattern when running at 5 m s^−1^. All participants wore minimalist shoes (Xero Shoes, Boulder, CO, USA) to maintain consistency in footwear and allow for exposure of the dorsal aspect of the foot for accurate marker placement and identification of foot strike patterns.

For this study, marker trajectories, ground reaction force, dynamic ultrasound images and muscle activity were collected. All experimental data were synchronized via a digital output signal generated by the ultrasound device that triggered the collection of the marker trajectories, ground reaction forces, and EMG signals. Marker trajectories were collected using an eight-camera motion capture system (Qualisys, Gothenburg, Sweden) sampling at 250 Hz. Small retro-reflective markers (6–14 mm) were placed on the upper- and lower-limbs of the participant as detailed in Lai et al.^14^. Ground reaction force was collected using an instrumented treadmill (Tandem Treadmill, Advanced Mechanical Technology, Watertown, MA) sampling at 2000 Hz. Marker trajectories and ground reaction forces were filtered using a fourth-order, low-pass Butterworth filter with cut-off frequencies of 20 Hz and 60 Hz, respectively. Ground reaction forces were filtered at a higher cut-off frequency to preserve the majority of the external impact signal but remove the majority of the measurement noise.

Muscle activity of the SO was measured using electromyographic (EMG) signals collected through a telemetered system (Noraxon, Scottsdale, AZ, USA) sampling at 2000 Hz. Ag/AgCl surface electrodes (20 mm inter-electrode distance) were placed on the belly of the muscles according to SENIAM (Surface Electromyography for Non-Invasive Assessment of Muscles) recommendations^22^. The information contained in the raw EMG signals were quantified using EMG specific wavelet analysis techniques^23^. Specifically, following the protocol devised by von Tscharner^23^, a filter bank of 11 non-linearly scaled wavelets, with parameters set to ensure the original SO EMG intensities could be approximately reconstructed from the sum of the wavelet transformed signals, were defined to represent a band-pass filter for the signal spanning 6.90–395.44 Hz^23^. The intensity from the first wavelet was not used in calculation of the total intensity, to mitigate the influence of motion artifacts on the EMG signal.

Dynamic 2D ultrasound images were collected using a PC-based B-mode ultrasound system (Telemed Echo Blaster 128, Vilnius, Lithuania) sampling at ~ 80 Hz. A flat profile 7 MHz ultrasound transducer with a scanning width and depth of 60 and 50 mm, respectively, was positioned at the mid-belly of LG at the midline of the mediolateral and proximodistal planes; perpendicular to the superficial and deep aponeuroses of SO where clear fascicle striation patterns were observed. The transducer was secured with self-adhesive bandages to reduce its displacement and to mitigate the influence of its weight on the in vivo oscillations measured in this study. The right leg was studied for all participants.

### Computational segmentation of ultrasound images

**Figure 1:**
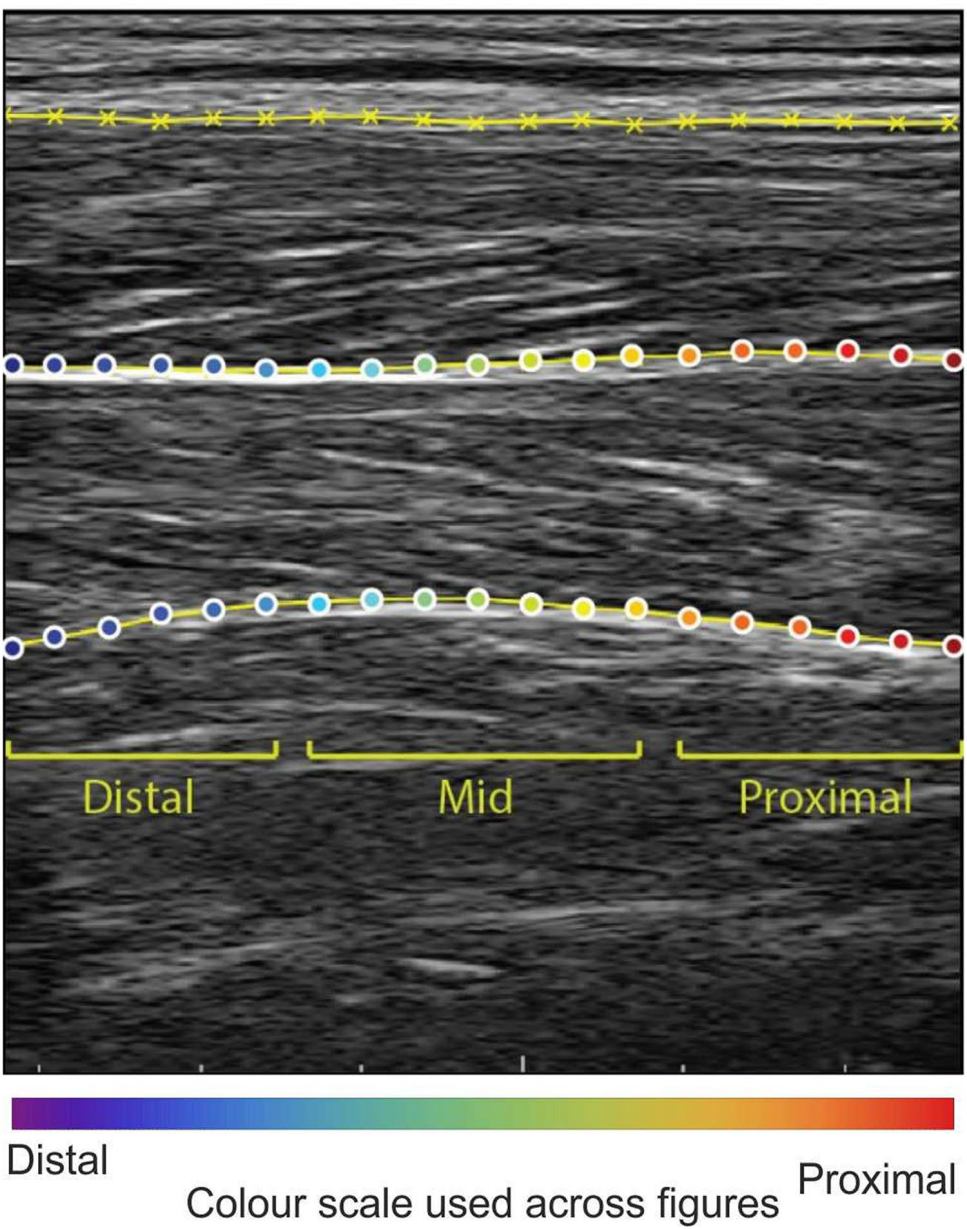
Exemplar ultrasound image with segmentation performed on the superficial and deep aponeuroses of the soleus (SO) muscle during stance phase of steady-state running at 5 m s^−1^. The distal, mid and proximal sections represent the anatomical regions of the points landmarks within the ultrasound image. Example of segmented ultrasound image sequences from this data set can be seen in^16^.

The point landmarks on the superficial and deep aponeurosis of the SO were segmented using an active shape model^18^ that has previously been shown to provide segmentations to within ~ 1 mm of manual labels^18^. Briefly, a points distribution model (PDM) was created for each participant that captured the variability in the shapes of the aponeurosis. Landmarks consisted of x- and y-coordinates of 19 evenly-spaced pixel points, 32 pixels separating each, that spanned the entire image width (Fig. 1). We first manually digitised landmarks for every 20^th^ frame of ultrasound image sequences taken from running at 3 and 4 m s^−1^. These two running conditions were chosen because they represented conditions with the largest variability in aponeurosis shapes. Unlike Darby et al.^18^, the training ultrasound images were isolated to the participant rather than across all the participants. This approach was used as it provided the best active shape model fit to each participant’s image set given the large displacements exhibited by the aponeurosis across the analysed locomotion conditions, especially at fast running.

To perform a probabilistic search for known aponeurosis shapes in the analysed images, a multi-resolution active shape model (ASM) was generated using augmented training shapes. Mean shape and covariance matrix, decomposed from the training shapes, were augmented using Gaussian models of image intensity surrounding each landmark. Intensity gradient of pixels adjacent to each landmark were calculated for multiple image resolutions (three image scales for all images except the first, where five were used to initialise the segmentation process; see Fig. A1 in Darby et al.^18^) to provide a coarse-to-fine search for solutions. The ASM was then passed over unsegmented ultrasound images and varied the PDM landmarks to accurately define the location of the landmarks in each image. The ASM iterated through new landmarks and predicted the likelihood the landmarks matched the shape of the aponeurosis by minimising the Mahalanobis distance (distance of a point to a distribution) with the pre-trained intensity model. Once the Mahalanobis distance was minimised for all landmarks, the new PDM was compared with previously defined shapes and the nearest shape (within three standard deviations) was chosen as the shape of the aponeurosis. The optimised PDM landmarks were then used as the initial guess for the next image frame.

Once all the images from the trials were segmented, they were visually inspected to ensure that the PDM landmarks fit the aponeuroses appropriately. One participant was removed from the study because the deep aponeurosis of the SO was not sufficiently distinct from the surrounding tissues and hence, we were not confident in the accuracy of the fitting approach. All presented data are therefore from *N* = 9 participants. This computational analysis approach resulted in the position and shape of the SO aponeuroses quantified in a total number of images ranging from ~ 450 to ~ 600 images per trial, with this information taken forward for further analysis. In total, ~ 37,000 images were processed.

### Data analysis

Data across four stance phases for each locomotion condition were processed for each participant. Due to the relatively low sampling frequency of the ultrasound images, we used the point landmark displacement during the entire stance phase to examine the characteristics of the in vivo oscillations through the SO. The point landmark transverse displacement, in the image frame of reference, represented the superficial and deep displacement of the tracking points relative to the skin-surface (or the location of the ultrasound probe). The transverse displacement was resampled to 100 data points per stance phase, averaged across participants and expressed as group means ± SEM. Point landmark displacements were normalised to equivalent point landmarks segmented from a static ultrasound image during quiet standing. Total EMG intensity of the SO was normalised to its peak EMG intensity measured across all locomotion conditions.

### Input signal frequency

To calculate the input signal to the body due to ground impact, we used the method described by Boyer et al.^6^. Briefly, the input signal frequency was determined from the vertical component of the ground reaction force. The rate of force development was computed as the time derivative of the vertical ground reaction force. The frequency of the input signal due to impact was estimated as the inverse of the time between maximal rate of force development and maximal impact force. This frequency represented a quarter of a whole wave oscillation of the impact signal.

### Power spectrum analysis to quantify muscle tissue transverse vibrations

We used a power–frequency analysis on the transverse displacement of the point landmarks, located on the superficial and deep aponeuroses of the SO, to quantify the power (relating to the size of transverse displacements) and frequency (relating to the changes in transverse displacement as a function of time) content of the in vivo transverse oscillations of the SO at each locomotion condition. To minimise low frequencies of movement-related displacement in deformable soft tissue, we filtered the displacement with a 4th order Butterworth high-pass filter with a cut-off frequency equal to stride frequency at each locomotion condition. The cut-off frequencies were 1.3, 1.8, 2.4, 2.6, 2.7, 2.9 and 3.1 Hz for steady-state walking speeds of 0.7, 1.4 and 2 m s^−1^ and running speeds of 2, 3, 4 and 5 m s^−1^, respectively. A power–frequency analysis, computed using fast Fourier transform (MATLAB, Mathworks Inc., Natick, MA, USA), quantified the power across a range of frequencies between 0 and 40 Hz for the transverse displacement of each point across the entire stance phase. From the power–frequency spectrum, we extracted the following variables: peak power and the cumulative frequency at which the area underneath the power–frequency curve was 50% of the total area. This cumulative frequency represented the frequency at which the accumulated power with respect to frequency from the in vivo oscillations was half of the total signal (i.e. the transverse displacement frequencies that accounted for half the signal power, or the oscillation frequencies that made up the majority of the signal), limited by the sampling frequency of the in vivo ultrasound measurements (~ 80 Hz).

### Statistical analysis

A one-way repeated measures ANOVA was conducted using R (v.3.5)^24^ to test for statistically significant differences in the input signal with locomotion condition. In addition, we defined three groups of point landmarks based on their location within the ultrasound image: distal, mid and proximal. Point landmarks 2–6 represented the distal region, 8–12 represented the mid region, and 14–18 represented the proximal region (Fig. 1). Two-way repeated measures ANOVA was conducted to test significance of peak power and cumulative frequencies of the point landmark transverse displacement on each aponeurosis (analysed independently) with image region and locomotion condition defined as main effects. If significant main effects were observed, Bonferroni-corrected post-hoc tests were performed to test pairwise comparisons. A *p* value of 0.05 was set as significance.

## Results

**Figure 2:**
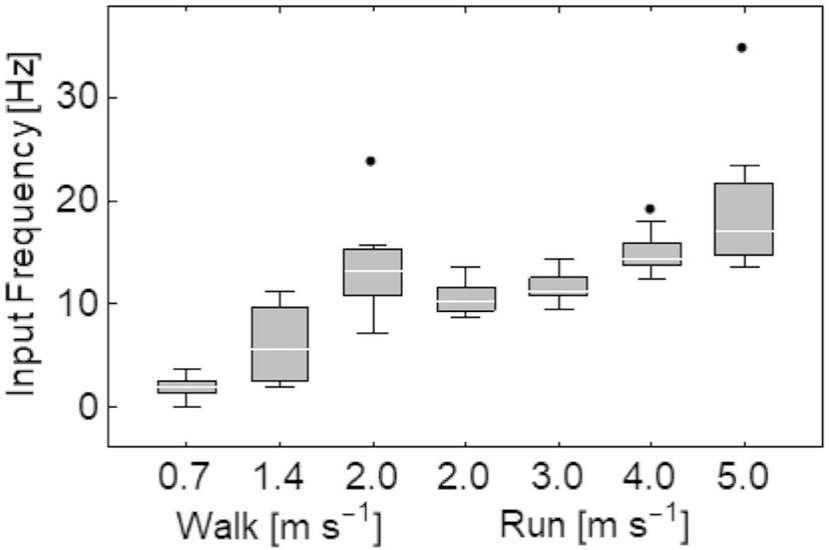
Input frequency determined from the vertical ground reaction force vector during stance phase across walking and running at seven steady-state speeds. The input frequency represents a quarter of a whole wave oscillation during the impact phase of stance phase. Box plots display the mean with the lower and upper box edges representing 25% and 75% quantile values respectively. Outlier values are denoted by solid circles, and denote points lying outside the upper quartile plus 1.5 the inter-quartile range.

**Figure 3:**
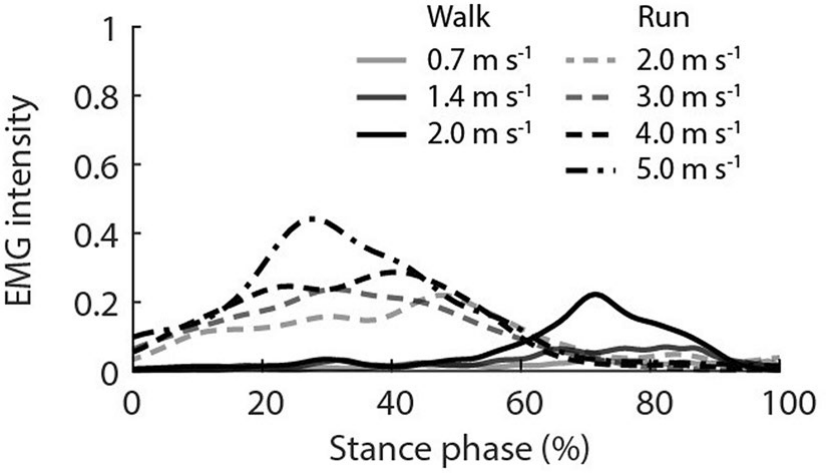
Total EMG intensity of the soleus muscle during stance phase across walking (solid lines) and running (dashed lines) at seven steady-state speeds.

The signal frequency of the impact, quantified from the ground reaction forces, significantly increased with both increased walking and running speeds (*p* < 0.001; Fig. 2). The frequency was slightly higher during walking at 2 m s^−1^ (13.6 ± 1.6 Hz) compared with running at the same speed (10.6 ± 0.5 Hz), however, a post-hoc comparison between the two conditions was not significant (*p* = 0.8). The SO showed periods of activity during stance phase of all locomotion conditions (Fig. 3) with peak EMG intensity increasing with faster walking speed during late stance phase and with faster running speeds during early-mid stance phase.

**Figure 4:**
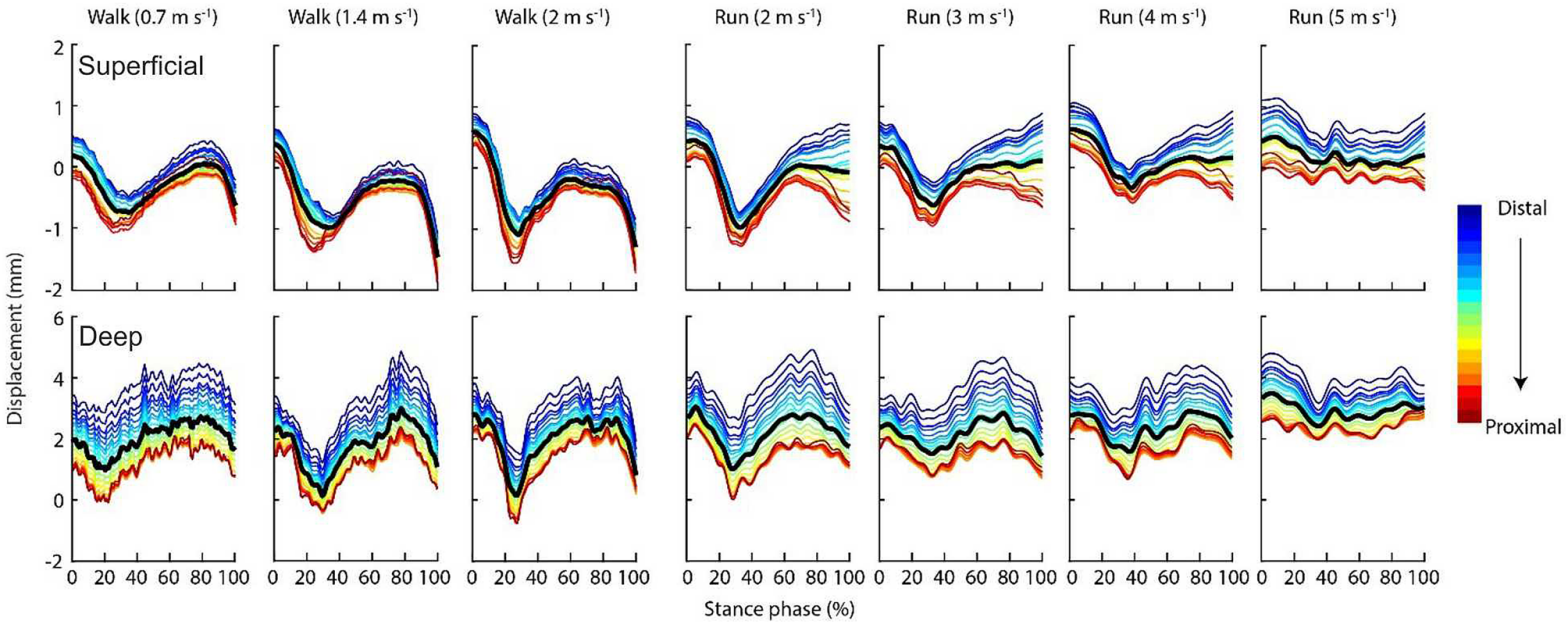
Transverse displacement of 19 point landmarks placed on the superficial (top) and deep (bottom) aponeuroses of the soleus muscle from the distal to proximal ends of the ultrasound image during stance phase of walking and running at seven steady-state speeds. The displacement was normalised to the position of the point landmarks during resting quiet standing. Negative and positive displacement represent deep (away from skin surface) and superficial (toward skin surface) movement, respectively. The black line denotes the mean.

The point landmarks on the superficial and deep aponeuroses of the SO underwent similar transverse displacement patterns during stance phase across locomotion conditions; though there were subtle differences between gait and speed conditions (Fig. 4). In general, all point landmarks exhibited an initial downwards displacement in the image (or deep in the anatomical reference frame) and then returned to the initial position during the second half of stance before shifting downwards (deep) again during push-off into swing phase. With increased walking speed, there was greater initial landmark transverse displacement on both aponeuroses during the first 40% of stance phase while with increased running speed, the point landmarks exhibited a reduction in peak-to-peak displacement. Furthermore, for both aponeuroses, point landmarks located towards the distal region of the muscle underwent a greater displacement from their position during static standing compared with those located towards the proximal region.

**Figure 5:**
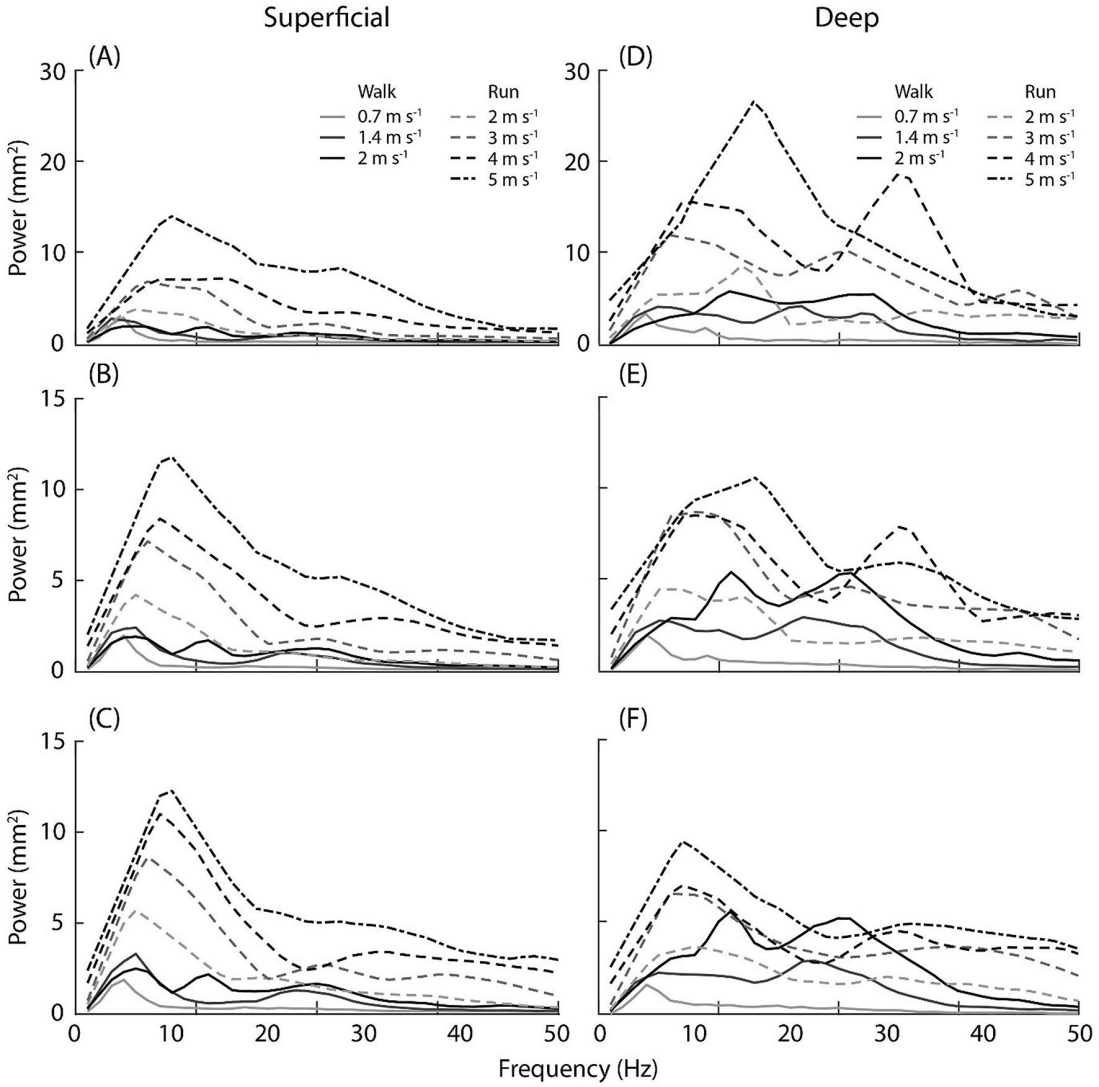
Power–frequency spectrum using the displacement of the point landmarks on the superficial (left) and deep (right) aponeuroses for the distal (**A**,**D**), mid (**B**,**E**) and proximal (**C**,**F**) tracking point regions during stance phase of walking (solid lines) and running (dashed lines) at seven steady-state speeds. Due to the relatively low sampling rate of the ultrasound measurements (80 Hz), the frequency spectrum analysed was only to 40 Hz, the scale here includes values up to 50 Hz (although it is outside the Nyquist frequency) to illustrate that the power–frequency tails off at higher frequencies.

**Figure 6:**
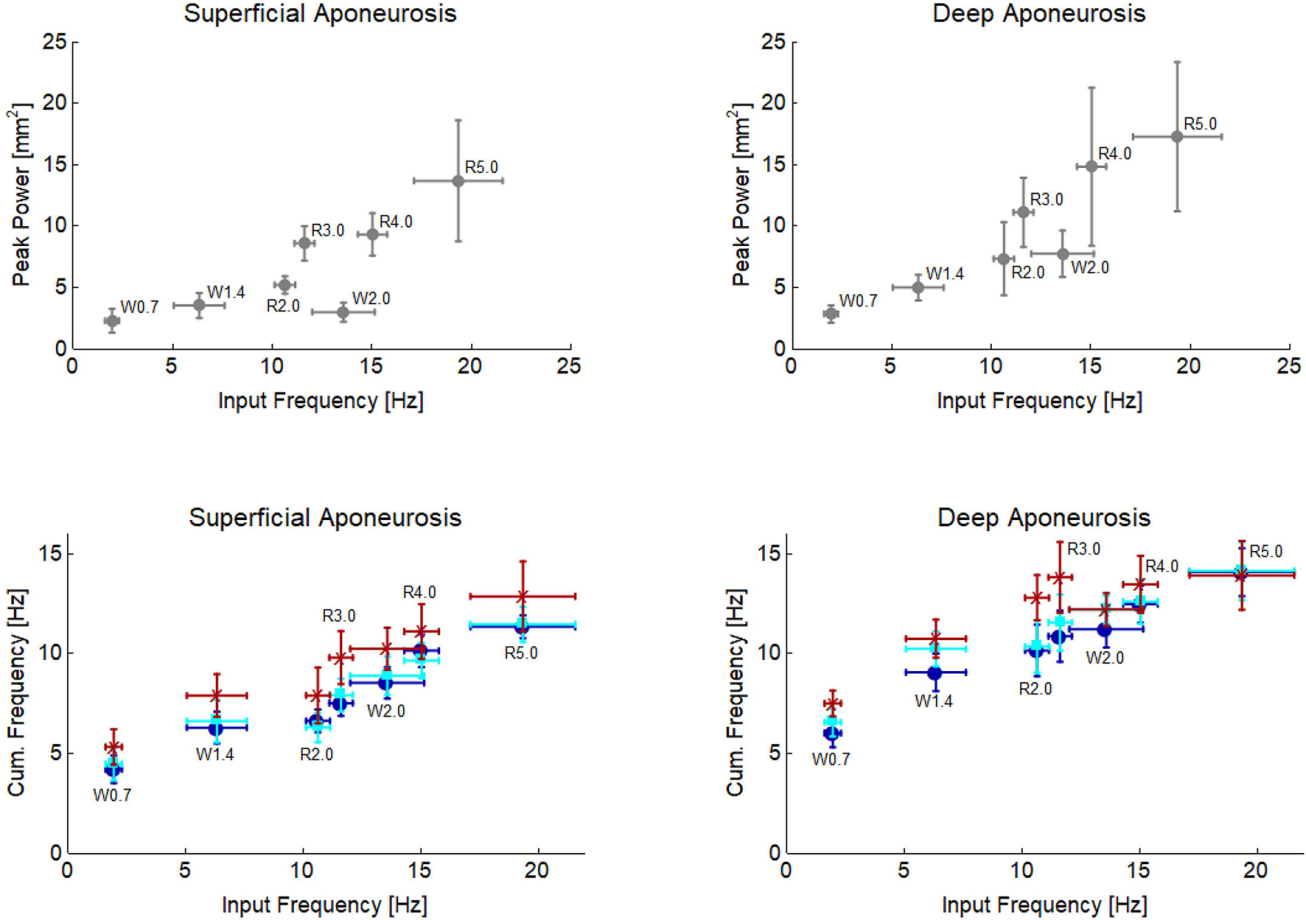
Peak power (top row) and cumulative frequency (bottom row) of superficial (left) and deep (right) soleus aponeurosis plotted as a function of input frequency. Cumulative frequency of the distal (dark blue circle), middle (cyan square) and proximal (dark red cross) image region are shown.

The power–frequency analysis performed on the displacement characteristics found significant main effects for both power and frequency content with locomotion condition and with image region (Fig. 5). Specifically, peak power of the displacement oscillations on superficial and deep aponeuroses significantly increased across locomotion conditions (*p* < 0.001;* p* = 0.002, respectively), where on average the increase was 2.8 mm^2^ with increased walking speed and 9.2 mm^2^ with increased running speed. This revealed a relationship between peak power and input frequency (Fig. 6). In the deep aponeurosis the relationship was well explained by a linear model, with *r*^2^ = 0.85 ($y=0.05+0.84x$). While in the superficial aponeurosis, the linear fit was reasonable (*r*^2^ = 0.65) but the relationship was better explained by a quadratic fit (*r*^2^ = 0.93, $y=2.86-0.18x+0.04{x}^{2}$).

For both superficial and deep aponeuroses, cumulative frequency had significant main effects with locomotion condition (both *p* < 0.001) and image region (*p* = 0.016;* p* = 0.043). Cumulative frequency increased on average two-fold with both increased walking speed from 0.7 to 2 m s^−1^ and with increased running speed from 2 to 5 m s^−1^. Due to the association of locomotion condition and input frequency (Fig. 2) a relationship with input frequency was also apparent (Fig. 6). Cumulative frequency also differed significantly between the regions for both aponeuroses; most noticeably between the distal and proximal regions where on average across locomotion conditions and aponeuroses, the cumulative frequencies differed by ~ 1.6 Hz.

## Discussion

Impact forces, caused by the foot contacting the ground during locomotion, can be considered an input signal that is expected to produce vibrations in the soft tissues of the body. To minimise injury risk and muscle fatigue^1^, it is important for the body to damp these input signals, and skeletal muscles have been proposed to play a role in this task. Previous measurements of soft tissue oscillations caused by locomotion-related input signals have however been restricted to skin mounted devices. Therefore, no direct observations of these behaviours in skeletal muscles in vivo have been reported. This study examined if 2D ultrasound imaging could be used to measure such oscillations through the muscle during locomotion. The results supported our hypothesis that faster walking and running speeds induce higher input frequencies, related to greater ground impact force that would have occurred within a shorter stance duration. Furthermore, over the observed increase in input signal frequency, greater power and frequency content were extracted from the transverse displacement of landmarks measured in vivo on the superficial and deep aponeuroses of the soleus muscle. Thus, this study provides the first evidence that ultrasound imaging can measure the influence of an external impact stimulus on in vivo muscle dissipative behaviours related to changes in locomotion type and speed.

The input frequencies of 10–20 Hz recorded here (Fig. 2) were consistent with previous studies that reported frequencies of 10–30 Hz during running at equivalent steady state speeds^1,9^. All studies report clear increases in input frequencies with faster walking and running. In agreement with these studies, we also found a positive association between input frequency and power and frequency content in soft-tissue oscillations. Previous studies reported oscillation frequencies of 14–40 Hz for artificially-induced vibrations during isometric contractions^1,25^ and ground contact during running across a range of steady-state speeds^6^. However, due to a number of factors related to different measurement methods, the lower cumulative frequencies reported here (~ 5–15 Hz, Fig. 6) should not be directly compared and are likely to provide different insights into in vivo tissue behaviour to consider. For example, systems that measured soft-tissue vibrations at the skin surface will encompass oscillations and interactions across several body tissues, whereas ultrasound-derived data aims to quantify muscle-specific oscillations. The use of 2D ultrasound imaging therefore provides opportunity to quantify tissue specific responses to locomotor conditions, particularly deeper tissues, that may not be taken from the amalgamated responses recorded by skin mounted devices (e.g. do activation patterns across different muscles lead to individual tuning to dissipate impact forces or is the neuromuscular response more homogeneous?). However, for ultrasound to be more widely applied to answer such questions, a number of key methodological points will need to be considered and are discussed below.

**Figure 7:**
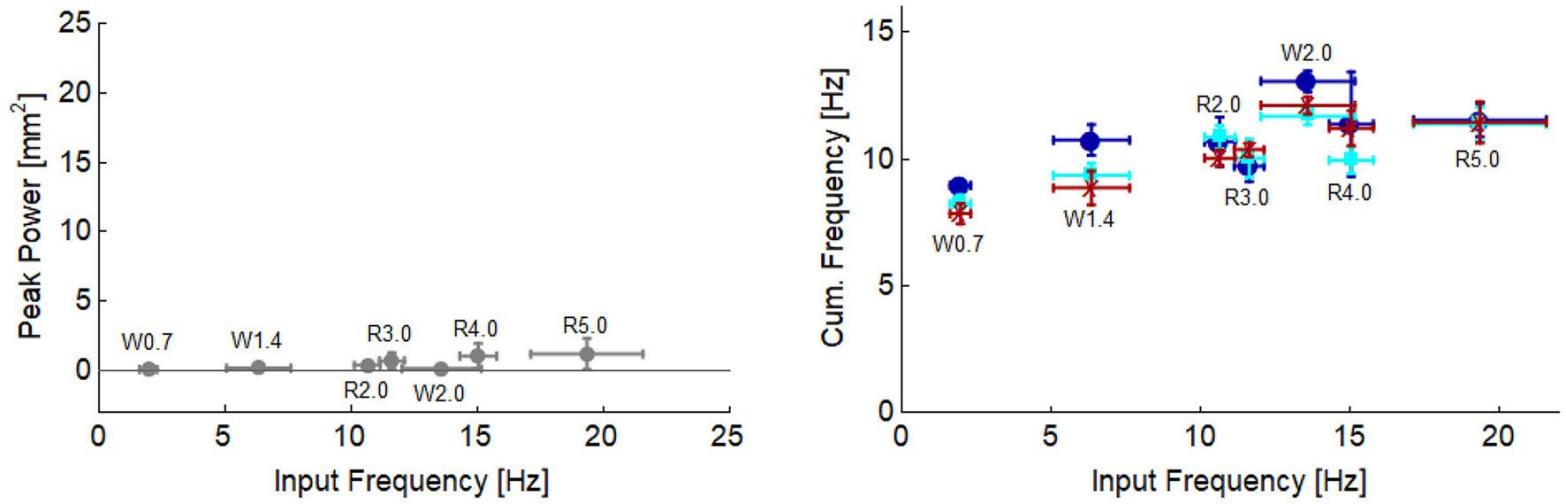
Peak power (left) and cumulative frequency (right) of superficial aponeurosis of lateral gastrocnemius plotted as a function of input frequency. Cumulative frequency of the distal (dark blue circle), middle (cyan square) and proximal (dark red cross) image region are shown. Values were calculated using same methods used for soleus data, reported in the methods.

With current technology, ultrasound-derived measures to study behaviour of soft-tissues will only be possible for deeper tissues. This relates to the current practice for obtaining high quality images during dynamic movement, involving the ultrasound probe being strapped and secured tightly to the skin surface. This compresses the muscles and reduces the amount of muscle bulging^26,27^, particularly in the superficial muscles (e.g. superficial aponeurosis of the medial and lateral heads of the gastrocnemius). When we ran the power–frequency analysis for the superficial aponeurosis of the LG (segmented and analysed in the same way as reported soleus data), we found very low peak power values (≤ 1.24 mm^2^) and the cumulative frequency was ≤ 13 Hz across all conditions (Fig. 7). This contrasts with the peak power and frequencies observed in the superficial and deep aponeuroses of the soleus (Figs. 5 and 6). It therefore seems clear that the strapping compression has influenced the oscillations measured here, leading to our decision to only report results for soleus and suggesting that 2D ultrasound-based approaches for measuring muscle specific oscillations may be limited to deep muscles only.

The effects of compression does however offer some useful insight into whether the muscle oscillations reported may just reflect jolting or oscillation of the probe on the skin surface during locomotion. If this were a major feature captured in the ultrasound measures, it would be expected that all image, and hence muscle, regions would be equally affected. Differences in oscillations recorded between the aponeurosis regions (Figs. 4, 6 and 7) as well as across aponeuroses (Figs. 4, 5, 6, 7) however show that this is not the case. Leading us to believe that reported measures represent the in vivo muscle behaviour rather than probe effects.

This study measured the transverse displacement and frequency of the superficial and deep aponeuroses of the soleus using ultrasound and computational image processing techniques to quantify in vivo muscle oscillations in the ankle plantar flexors. The analysis involved quantifying the transverse oscillations of the aponeuroses perpendicular to the direction of the impact. Ideally, quantifying oscillations in the transverse direction as well as the travelling wave oscillation in the longitudinal direction would provide a more complete description of how in vivo muscle respond to or are affected by different impact forces. Additionally, future studies that focus on the time domain rather than the frequency domain can utilise the displacement, velocity and acceleration of the aponeuroses points as the impact signal travels through the muscle and the ultrasound image. However, two factors need to be considered: (1) the analysis approach employed here assesses displacement in a fixed location of the image. As the muscle will translate through the image during the gait cycle, point displacements are not consistently quantified for a given muscle region; (2) the sampling rate and field of view provided by the ultrasound device will also significantly affect the oscillation characteristics that can be reliably quantified. For example, the ultrasound image sampling rate used here (~ 80 Hz) may not be sufficient to quantify oscillations, particularly in the longitudinal direction of the impact signal (i.e. as waves travel along the muscle length). The signal frequency of the impact during running at 5 m s^−1^ was on average 20 Hz (Fig. 2). The vibration characteristics of the input frequency will be at most contained in four ultrasound images, which made it difficult to quantify using the displacement approach described in this study and should be considered when reviewing the frequency bandwidth over which analyses could be completed (i.e. 0–40 Hz). Skin mounted accelerometer data do however indicate that, even for maximal sprinting, soft tissue vibrations do not contain components above 100 Hz, with the dominant frequencies occurring well below this (e.g. Figures 3 and 4 in Enders et al.^28^). The power spectra recorded with the ultrasound here also contain similar complexities (i.e. multiple peaks in deep aponeurosis Fig. 5) to those seen in accelerometer data^28^ highlighting commonalities that indicate the ultrasound-derived measures are physiologically related and that locomotion related soft-tissue vibrations should be considered more than a single sinusoidal oscillation^28^.

From the presented results, it is clear that within each gait increased locomotor speed is associated with increased input frequency and increased oscillation frequencies in both the deep and superficial aponeurosis of soleus. Interestingly, while input signal frequency was greater during walking compared to running at the same velocity, peak power was lower (superficial aponeurosis) or the same (deep aponeurosis) (2 m s^−1^, Fig. 6). This may reflect alterations in the EMG signal amplitude and timing (Fig. 3), as muscle activation patterns have been shown to tune muscle responses to impact forces^25^. Additionally, the foot-strike patterns could also have been influential. For example, during walking and in some people’s running gait initial ground contact occurs at the heel, so the impact could be directly transferred to the calf muscles. For foot contacts when the toes impact the ground before the heel, as occurs in some running, the impact could be influenced by ankle joint rotation before oscillations were observed in the soleus muscle. While we did not control for foot-strike patterns during running, to examine this possible influence on muscle oscillations, we compared the cumulative frequencies of the superficial soleus aponeurosis during stance phase of each running condition of two participants that exhibited different foot-strike patterns. We observed on average a ~ 5 Hz difference in cumulative frequencies between participants across running speeds (greater values in the rearfoot foot-striker) indicating that foot strike pattern may potentially have an influence on in vivo muscle oscillations.

## Conclusions

In this study, we combined ultrasound imaging with computational image processing techniques to measure the transverse displacement of the aponeuroses of the soleus muscle to quantify in vivo muscle oscillations during walking and running. We found that muscle transverse oscillations in the soleus increase incrementally with greater input signal frequency. Thus, 2D ultrasound imaging has the potential to be used to examine oscillations specific to individual skeletal muscles, located deeper within the leg, and how it responds to different impacts when external interfaces such as shoe material and ground surface properties are altered and optimised to reduce fatigue and injury risk.

## Data Availability

Input frequency, peak power and cumulative frequency values for each participant and condition are available on Manchester Metropolitan E-space repository for readers to access. Please contact the corresponding author for the DOI.
